# Misclassification of child body mass index from cut-points defined by rounded percentiles instead of Z-scores

**DOI:** 10.1186/s13104-017-2983-0

**Published:** 2017-11-28

**Authors:** Laura N. Anderson, Sarah Carsley, Gerald Lebovic, Cornelia M. Borkhoff, Jonathon L. Maguire, Patricia C. Parkin, Catherine S. Birken

**Affiliations:** 1 Department of Health Research Methods, Evidence, and Impact, McMaster University, 1280 Main Street West, Hamilton, ON L8N 3Z5 Canada; 2 Child Health Evaluative Sciences, The Hospital for Sick Children Research Institute, Peter Gilgan Centre for Research & Learning, 686 Bay Street, Toronto, ON M5G 0A4 Canada; 3 The Applied Health Research Center of the Li Ka Shing Knowledge Institute of St. Michael’s Hospital, 209 Victoria St, Toronto, ON M5B 1T8 Canada; 4 Department of Nutritional Sciences, Faculty of Medicine, University of Toronto, FitzGerald Building, 150 College Street, Room 316, Toronto, ON M5S 3E2 Canada; 5 Department of Pediatrics, Faculty of Medicine, University of Toronto, 555 University Avenue, Toronto, ON M5G 1X8 Canada; 6 Institute for Health Policy, Management, and Evaluation, University of Toronto, Health Sciences Building, 155 College Street, Suite 425, Toronto, ON M5T 3M6 Canada; 7 Division of Pediatric Medicine, The Hospital for Sick Children, 555 University Avenue, Toronto, ON M5G 1X8 Canada

**Keywords:** Growth charts, Body mass index, Pediatric obesity, Child, preschool, Validation studies

## Abstract

**Objective:**

To evaluate the misclassification resulting from the use of body mass index (BMI) cut-points defined by rounded percentiles instead of Z-scores in early childhood. Using data from the *TARGet Kids* primary care network we conducted a cross-sectional study among 5836 children < 6 years of age. The World Health Organization growth standards were used to calculate BMI-for-age Z-scores. BMI Z-score cut-points of < − 2.0, > 1.0, > 2.0, > 3.0 are recommended to define wasted, at risk of overweight, overweight and obese. However, rounded percentiles of the 3rd, 85th, 97th, and 99.9th are commonly used. Misclassification was calculated comparing the frequency distributions for BMI categories defined by rounded percentiles and Z-score cut-points.

**Results:**

Using rounded percentiles, the proportion of children who were wasted, at risk of overweight, overweight, and obese was 4.2, 12.5, 4.3 and 0.8%, whereas the distribution using Z-scores was: 3.6, 13.8, 3.4 and 1.0%, respectively. Overall, 117 (2%) children were misclassified when using percentiles instead of Z-scores; however, 13% (33/245) of children who were wasted and 14% (8/57) of children who were obese were misclassified. Misclassification of child growth results from the use of cut-points defined by rounded percentiles instead of Z-scores and limits comparability between studies.

*Trial registration* Clinicaltrials.gov NCT01869530 June 5, 2013

## Introduction

Monitoring of child growth, including classification of both underweight and overweight categories, is important for population health, and routine child growth monitoring is recommended [1]. The World Health Organization (WHO) Child Growth Standards charts are recommended as a reference standard for the growth monitoring of children 0–5 years of age and have been endorsed by many countries [2, 3]. The WHO Growth Standards were developed following children from 6 countries under optimal growth conditions [4]. Regardless of which reference standard is used, cut-point definitions are required for clinicians and researchers to classify children’s growth status. These cut-points for age and sex standardized growth measures are defined using either Z-scores or percentiles.

The WHO Child Growth Standards provide child growth measures standardized by age and sex using Z-scores. Z-scores, or standard deviation scores, describe where an observation falls within a number of standard deviations of the mean. For example, body mass index (BMI)-for-age Z-score cut-points of < − 2.0, > 1.0, > 2.0 and > 3.0 are recommended by the WHO to classify children 0–5 years of age as wasted, risk-of-overweight, overweight, and obese, respectively [5]. Several advantages of the Z-score for population-based assessment of child growth have been described [6]. Z-scores are on a linear scale, with the same interval between values across the distribution; this allows for calculation of the mean and standard deviation [7].

In contrast, percentiles rank a child’s position in comparison to a reference population. Z-scores can be converted directly to percentiles [8], although when converted the percentiles are often rounded. Rounded percentiles of the 3rd, 85th, 97th and 99.9th are commonly used in clinical practice and recommended for monitoring child growth in Canada [2]. These rounded percentiles correspond to exact Z-scores of − 1.88, 1.04, 1.88 and 3.09, in comparison to the WHO recommended cut-points of − 2.0, 1.0, 2.0 and 3.0, respectively. Z-scores have been recommended for research, and percentiles for clinical settings as they may be easier to understand [7].

However, the use of cut-points defined by rounded percentiles instead of exact z-scores may result in misclassification of children’s growth status, yet to the best of our knowledge, the magnitude of this misclassification has not been evaluated. This may be important when comparing differences between studies that have unintentionally used slightly different cut-points. The objective of this study was to evaluate the degree of misclassification resulting from the use of cut-points defined by rounded percentiles relative to Z-scores for growth monitoring in early childhood.

## Main text

### Study design

A cross-sectional study was conducted among children 0–5 years. Children were recruited from scheduled well-child primary care visits through TARGet Kids!. TARGet Kids! is a primary care practice based research network (http://www.targetkids.ca) [9, 10]. Children were recruited from nine primary care pediatricians or family practice clinics in Toronto, Canada between 2008 and 2016. Children with severe developmental delay or chronic illness (except for asthma) were excluded.

### Anthropometric measures

Trained research team members measured child weight and height (or length for children < 2 years). Weight was measured using a precision digital scale (SECA, Germany). A stadiometer was used to measure standing height (SECA) for children 2 years of age and older and length was measured for children under 2 years of age using a length board [9]. Weight in kilograms was divided by the height (or length) in meters squared to calculate BMI.

Age and sex standardized BMI Z-scores were defined using the World Health Organization (WHO) growth standards [11]. The WHO growth standards were selected as they are meant to reflect optimal growth in children and are recommended for use in this age group in Canada [2]. BMI-for-age Z-score cut-points of < − 2.0, > 1.0, > 2.0 and > 3.0 were used to classify children as wasted, risk of overweight, overweight and obese, respectively [2]. BMI-for-age Z-scores were categorized using both the Z-score cut-points of < − 2.0, > 1.0, > 2.0 and > 3.0, and rounded percentile cut-points of 3rd, 85th, 97th and 99.9th, which correspond to exact Z-scores of − 1.88, 1.04, 1.88 and 3.09.

### Statistical analysis

Cross tabulations of the number of children for each category were calculated comparing the two definitions (Z-score cut-points and rounded percentile cut-points) and discordant and concordant categories were reported. The percent misclassified was calculated both overall from the total and within growth categories. The relative percent misclassified within each category was calculated by dividing the difference between the two definitions (% defined by percentile minus Z-score cut-point) by the total percent defined by the Z-score cut-point. Kappa and weighted Kappa statistics were calculated as a measure of agreement. Although BMI is standardized for age and sex using the WHO growth standards, we further evaluated whether there were differences in misclassification by sex using stratified analysis.

## Results

There were 5836 unique children who participated in this study. The mean age of children was 28 months (SD = 19), 2770 (48%) were female and 3066 (53%) were male. The distribution of maternal ethnicity was 3818 (67%) European, 361 (7%) East Asian, 544 (10%) South or South East Asian, 277 (5%) African and 580 (11%) other or mixed ethnicity. The median neighborhood household income was $55,038 (SD = $25,484).

**Fig. 1:**
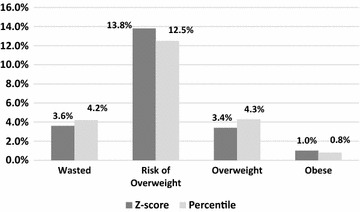
Comparison of BMI-for-age categories defined using percentile versus Z-score cut-points for child BMI Z-score (n = 5836). *The relative percent misclassification for wasted, risk of overweight, overweight and obese was 17, − 9, 26 and − 20%, respectively. Calculated as (percentile − Z-score)/Z-score

A total of 117 (2%) children were misclassified when using percentiles instead of Z-scores (Table 1). Within the extreme growth categories 13% (33/245) of children who were wasted and 14% (8/57) of children who were obese were misclassified by the use of rounded percentiles instead of Z-scores. Further, the *relative* percent misclassified for wasted, risk of overweight, overweight and obese was 17, − 9, 26 and − 20%, respectively, when using percentiles instead of Z-score cut-points (Fig. 1). Agreement between the two methods was high with a Kappa coefficient of 0.95 (95% CI 0.94, 0.96) and weighted Kappa of 0.96 (95% CI 0.95, 0.97).

**Table 1:** Agreement between growth categories using Z-score and percentile cut-points for classification of BMI categories in children 0–5 years of age (n = 5836)

Z-score cut-points	Percentile cut-points				
	Wasted	Normal	Risk of overweight	Overweight	Obese
	< 3rd	3rd to 85th	> 85th to 97th	> 97th to 99.9th	> 99.9th
< − 2.0	212 (3.6%)	0	0	0	0
− 2.0 to 1.0	33 (0.6%)	4530 (77.6%)	0	0	0
> 1.0 to 2.0	0	37 (0.6%)	727 (12.5%)	39 (0.7%)	0
> 2.0 to 3.0	0	0	0	201 (3.4%)	0
> 3.0	0	0	0	8 (0.1%)	49 (0.8%)

The results were similar when boys and girls were examined separately; a total of 68 (2.2%) boys and 49 (1.8%) girls were misclassified. The overall misclassification was slightly greater in boys than girls and this may be explained by the fact that more boys than girls were classified in the extreme growth categories. For example, using the Z-score cut-points, the percent of boys classified as wasted, risk of overweight, overweight and obese was 4.0, 14.4, 4.0 and 1.3% respectively, whereas the percent of girls was 3.1, 13.0, 2.8, 0.7%.

## Discussion

In this study of 5836 children 0–5 years of age we found misclassification from the use of zBMI cut-points defined by rounded percentiles instead of Z-scores. The overall degree of misclassification was small at only 2% of all children. However, within the extreme growth categories there was substantial misclassification, for example among children classified as wasted an additional 17% were misclassified, and among children classified as obese 20% were missed. Strengths of our study include the large sample size and standardized measurement of heights/lengths and weights by trained research assistants.

Recent population-based reports have used inconsistent cut-point definitions which we have shown may limit comparability for the extreme growth categories. For example, some studies have used percentile cut-points [12, 13], whereas in other studies Z-score cut-points have been used [14, 15]. It is possible that researchers are using Z-score cut-points in analysis and labeling them as percentiles for perceived ease of interpretation but this is not clear. We suggest that the terms not be used interchangeably or assumed that results would be the same.

Percentiles may be useful when interpreting values for individual children for both parents and health professionals, however, the WHO recommends that researchers use Z-scores for consistency and comparability. The use of percentiles also causes problems due to limited range especially at the extreme ends for example one proposed definition of severe obesity in young children is 1.2 times the 95th percentile. Due to the misclassification that results when cut-points defined using rounded percentiles and Z-scores are compared and the limitations of percentiles, we suggest that future data analysis is performed using Z-score cut-points and not percentiles [8]. To the best of our knowledge, we are the first to evaluate the misclassification from rounded percentiles versus Z-score cut-points.

## Limitations

One limitation of this study is that we were not able to evaluate the validity of each approach in relation to a gold-standard. We were unable to evaluate the clinical implications of this misclassification. Further, we were unable to estimate how frequently this misclassification occurs in the literature as it is often not clear which approach was used in previous studies. The choice of cut-points may not matter when comparing repeated measures over time within one population or within one child, but the observed differences may be very important when comparing the prevalence of growth categories across different studies or populations (e.g., for public health surveillance). Others may argue for the use of continuous variables only due to inherent limitations of the use of any cut-points such as reduced power and loss of information. However, cut-points are often useful when interpreting results, defining high-risk individuals and are frequently used for both research and for clinical purposes. The validity and reliability of BMI-for-age in early childhood have been evaluated elsewhere [16–18].

Although the overall degree of misclassification from the use of cut-points defined by rounded percentiles compared to Z-scores was small, substantial misclassification was observed within the extreme growth categories. This misclassification may have important implications when comparing both prevalence estimates for population surveillance and risk estimates from research studies. This misclassification may substantially impact on policy and planning. Future guidelines should clearly recommend the use of either Z-scores or rounded percentiles for consistency in child growth monitoring.

## Abbreviations

BMI: body mass index
WHO: World Health Organization
